# Evaluation of a calibration rig for stereo laparoscopes

**DOI:** 10.1002/mp.16310

**Published:** 2023-02-22

**Authors:** Thomas Dowrick, Guofang Xiao, Daniil Nikitichev, Eren Dursun, Niels van Berkel, Moustafa Allam, Bongjin Koo, Joao Ramalhinho, Stephen Thompson, Kurinchi Gurusamy, Ann Blandford, Danail Stoyanov, Brian R. Davidson, Matthew J. Clarkson

**Affiliations:** ^1^ Wellcome EPSRC Centre for Interventional and Surgical Sciences UCL London UK; ^2^ Royal Free Campus UCL Medical School Royal Free Hospital London UK

**Keywords:** calibration, image‐guided surgery, laparascope, stereo

## Abstract

**Background:**

Accurate camera and hand‐eye calibration are essential to ensure high‐quality results in image‐guided surgery applications. The process must also be able to be undertaken by a nonexpert user in a surgical setting.

**Purpose:**

This work seeks to identify a suitable method for tracked stereo laparoscope calibration within theater.

**Methods:**

A custom calibration rig, to enable rapid calibration in a surgical setting, was designed. The rig was compared against freehand calibration. Stereo reprojection, stereo reconstruction, tracked stereo reprojection, and tracked stereo reconstruction error metrics were used to evaluate calibration quality.

**Results:**

Use of the calibration rig reduced mean errors: reprojection (1.47 mm [SD 0.13] vs. 3.14 mm [SD 2.11], *p*‐value 1e−8), reconstruction (1.37 px [SD 0.10] vs. 10.10 px [SD 4.54], *p*‐value 6e−7), and tracked reconstruction (1.38 mm [SD 0.10] vs. 12.64 mm [SD 4.34], *p*‐value 1e−6) compared with freehand calibration. The use of a ChArUco pattern yielded slightly lower reprojection errors, while a dot grid produced lower reconstruction errors and was more robust under strong global illumination.

**Conclusion:**

The use of the calibration rig results in a statistically significant decrease in calibration error metrics, versus freehand calibration, and represents the preferred approach for use in the operating theater.

## 1 INTRODUCTION

Laparoscopic camera calibration is a prerequisite for surgical image guidance systems that utilize model to image registration.^1^, ^2^, ^3^ The calibration accuracy is a key contributor to the overall system accuracy.^4^ In the simplest case, camera calibration consists of intrinsic calibration of the laparoscope cameras to determine the cameras' optical characteristics, enabling both the reconstruction of visible surfaces and the projection of virtual objects onto the image plane.^5^ Where multiple cameras are present, for example, stereo laparoscopes, it may also be necessary to perform an extrinsic calibration to determine the relative locations and orientations of the two lenses to enable stereoscopic reconstruction^6^ and reprojection. Finally, for systems that utilize external tracking systems such as electromagnetic or optical systems,^7^ it becomes necessary to perform a hand–eye calibration to determine the location and orientation of each camera relative to the reference frame. The term hand–eye derives from the methods' origin in the robotics literature^8^, ^9^ where the camera forms the robot's eye and the hand refers to the robotic end‐effector holding the camera.

Calibration of surgical laparoscopic cameras brings further challenges including the requirement to maintain sterility, constraints on the range of laparoscope motion, the need to fit in with clinical workflows, and the requirement to be performed by clinically trained theater staff rather than technical specialists. These challenges, along with the basic mathematical challenges, mean that although the literature on laparoscope calibration is extensive, we still lack a standard, repeatable methodology for performing this essential operation. Safe clinical practice is dependent on the existence of clear protocols that describe how a procedure should be performed and specific success criteria. This has led us to develop a calibration rig and protocol that enables robust and repeatable calibration by theater staff.

### 1.1 Background

Previous studies have investigated the main mathematical, image processing, and technical aspects of camera calibration. Some have gone further to propose methods that are compatible with the operating theater. However, in most cases, validation and comparison with alternative approaches are performed by the authors without clearly defining the acquisition protocols used. Although calibration procedures claiming to be fully automatic^8^, ^10^ are not new, they remain susceptible to variation in data application. The result is calibration procedures that are highly user‐dependent, which is not acceptable for an approach that is intended for wider clinical deployment.

In the computer‐assisted surgery literature, most authors use Zhang's method^11^ to determine the intrinsic parameters of a given camera. Zhangs's method gathers multiple images of a calibration pattern of known geometry and estimates the pinhole camera model that best models the projection of the calibration pattern to the captured images. Zhang's method has been shown to be fast and computationally stable; however, several researchers have developed ways to improve upon it. Most of these approaches focus on the algorithm used for feature detection of the calibration pattern. Zhang's original method uses a chess board (alternating black and white squares) and corner detection algorithms to locate the corners on each captured image. Datta et al.^12^ showed that the centres of a pattern of rings can be more accurately located than the chessboard corners.

A common drawback of simple chessboards or Datta's ring pattern is that the individual markers are not uniquely identifiable, the order of points can only be determined if the entire calibration pattern is visible. This restricts the range of views that can be used for calibration, making it harder to capture distortion near the camera's periphery. One way around this is to use uniquely identifiable features for the calibration pattern. Liu^13^ demonstrated this approach using the rdCalib marker pattern. Thompson et al.^14^ showed that using AprilTags^15^ removed the problems with distortion at the periphery; however, the feature detection is not as accurate as when using rings. More recently, ArUco^16^ tags have been combined with chessboards, to create ChArUco boards. Detectors for chessboards and ChArUco boards are implemented within the OpenCV^17^ libraries. Whilst the best approach for intrinsic calibration will depend on the users' exact needs, we have previously found that the choice of calibration pattern has very little impact on overall calibration accuracy.^14^

For systems with multiple cameras (stereo laparoscopes) or with tracking markers attached, the next stage of stereo and hand‐eye calibration is also a much studied problem. These calibrations are closely related and involve determining the geometric relationships between different cameras and tracking markers. Both stereo and hand‐eye calibration reduce to fitting a geometric model to two sets of pose estimates. For stereo calibration, these are the camera poses, usually estimated using a homography,^18^ whereas for hand‐eye calibration, one set of poses comes from the external tracking system.

Because different methods of pose estimation have different characteristic errors, different optimization methods are useful in different applications. For stereo calibration, we have chosen the method implemented with OpenCV,^17^ which performs an initial linear estimate, followed by bundle adjustment using the Levenberg Marquardt method, resulting in a solution that is a local minimum of a least‐squares error function between 2D image points, and their corresponding 3D projections.

For hand–eye calibration, the distribution of tracking errors will be heavily dependent on the tracking system used^7^ and is unlikely to fit the assumptions of isotropy, normality, and independence^19^, ^20^, ^21^ assumed by most optimization algorithms. For optical tracking systems, the effect of these errors is amplified by the length of the laparoscope. These mathematical challenges, together with the challenges of fitting into a clinical workflow mean that hand–eye calibration for laparoscopes remains an unsolved problem.

Jiang^22^ provides an extensive review of the research to date for hand–eye calibration in general. Some interesting examples relevant to clinical calibration include Lee et al. who shows that it is better to move the calibration pattern^23^ than the laparoscope, and that it is better to find the camera‐to‐laparoscope marker after the pattern‐to‐marker transformation.^24^ Song et al.^25^ examined the mathematics of the hand–eye optimization to identify situations in which singularities may occur and proposed new formulations to avoid them. For robotic systems where the remote centre of motion is known, Pachtrachai et al.^26^ have shown that building this constraint into the calibration algorithm hand–eye calibration can give improved calibration results. The same approach may be applicable to a calibration utilizing a rig.

Other recent approaches attempt to simplify the theater calibration problem to just hand–eye, enabling the use of markers that are easier to keep sterile,^27^, ^28^, ^29^ or indeed no markers at all.^30^, ^31^, ^32^, ^33^ Shao et al.^34^ expanded on this by developing a progressive hand–eye calibration that gives live feedback on expected calibration accuracy. Jackson et al.^35^ have recently demonstrated that hand–eye calibration of an optically tracked laparoscope can be achieved by imaging a second optically tracked stylus. These approaches have the drawback of requiring a prior calibration of the laparoscopes intrinsic and stereo parameters and also have not demonstrated the strict protocols that are necessary to make calibration not only practical, but verifiable and repeatable.

It has been previously shown that calibration methods are affected by the choice of camera positions used to capture data, and it is not clear what data will lead to a good calibration.^36^ Kang et al.^37^ identified some of the challenges with sterility that occur within the operating theater. Because of the need for sterility, optical tracking markers are usually attached to the laparoscope immediately before surgery, limiting the time and space available for calibration. Whilst many of the methods discussed attempt to address this problem in some way, none of them provide the standardized, repeatable calibration approach needed for clinical work. Providing a calibration rig and the protocol to go with it should reduce the variability of the clinical calibration procedure.

### 1.2 Clinical requirements

Any calibration procedure/equipment that is to be used in a surgical environment should (a) provide a calibration accuracy that is within the requirements of the entire registration pipeline, (b) be repeatable, providing consistent results across multiple calibrations, and (c) sterilizable.

While there has been no published analysis of the required accuracy from a clinical perspective, a typical target for tumor margins during resection is 10 mm, which places an upper bound on the accuracy requirements of the entire system. The total error will be made up of errors in calibration, tracking, and in the registration algorithm. While absolute errors in tracking marker localization are typically sub millimeters (the datasheet for the NDI Vega used in this study gives a 0.15‐mm RMS accuracy), the lever‐arm effect across the length of the laparoscope can amplify this up to 3 mm.^7^ Registration errors on a phantom can be <3 mm,^4^ but increase to 5–30 mm when evaluated on surgical data.^2^, ^4^ Taking this into account, it is reasonable to anticipate that calibration errors would have to be <3 mm, and ideally as low as possible, to keep the total error within the surgical margin.

### 1.3 Contribution of this paper

The main contributions are:

– We have developed a calibration rig that can sit on any flat stable surface in the operating room, such as a surgical trolley. The rig holds the laparoscope stationary while enabling the calibration pattern to be moved through a range of motions to achieve a good calibration, without hand tremor.
– We have chosen a protocol that is sufficient, and can be performed quickly by clinical staff after minimal training.
– We evaluate the accuracy in a fair manner. Error metrics, such as the reprojection error, are typically presented by projecting the same data as was used in calibration.^8^, ^10^, ^19^ This leads to an overly optimistic assessment of performance. It is more valid to use separate data‐sets for calibration and evaluation. This is equivalent to precalibrating the camera intrinsics on one data set, and optimizing hand–eye errors on another, which is also more reflective of the errors that will occur in “real life” data, where calibration is performed once at the start of surgery.

In our work, we use a Viking 3DHD laparoscope (http://www.conmed.com/), so all the results shown are on a stereo laparoscope. This paper does not present any methodological advancement particular to stereo laparoscopes, and the methods are equally applicable to mono laparoscopes.

## 2 METHODS

**FIGURE 1:**
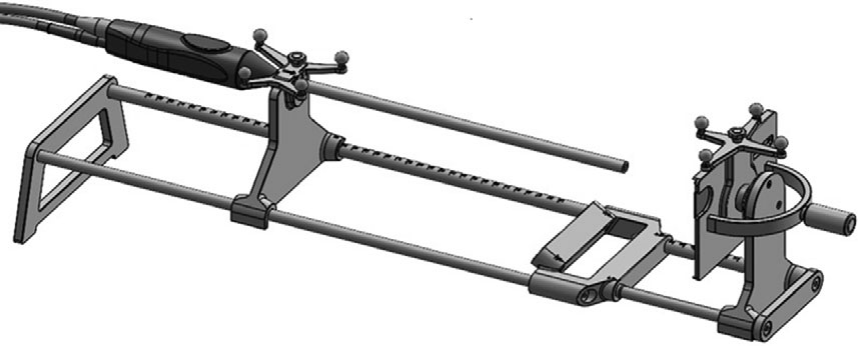
Calibration rig. Both the laparoscope and calibration target are held securely in place by the rig. The handle on the back of the target allows the user to rapidly and reproducibly adjust the angle of the target relative to the camera. Optical tracking targets are fixed on the laparoscope clamp, and the calibration target.

A custom calibration rig (Figure 1) was designed specifically to aid in laparoscope calibration.

– The laparoscope can be fixed securely in place with an adjustable thumbscrew.
– The calibration target is magnetically mounted, allowing its angle to be rotated via a handle on the back.
– Optical tracking targets for the scope and calibration pattern are built into the rig in fixed positions.
– Sliding rails allow the distance between the scope and the target to be adjusted.
– The angle at which the calibration target is attached to the main rails can be either 0°or 30°, depending on the laparoscope being used.
– Laser markings indicate the correct orientation of relevant parts, to aid new users in assembling the rig.
– All components are fabricated from stainless steel or titanium, for ease of sterilization.
– The rig itself, excluding the laparoscope, weighs approximately 3 kg.

**FIGURE 2:**
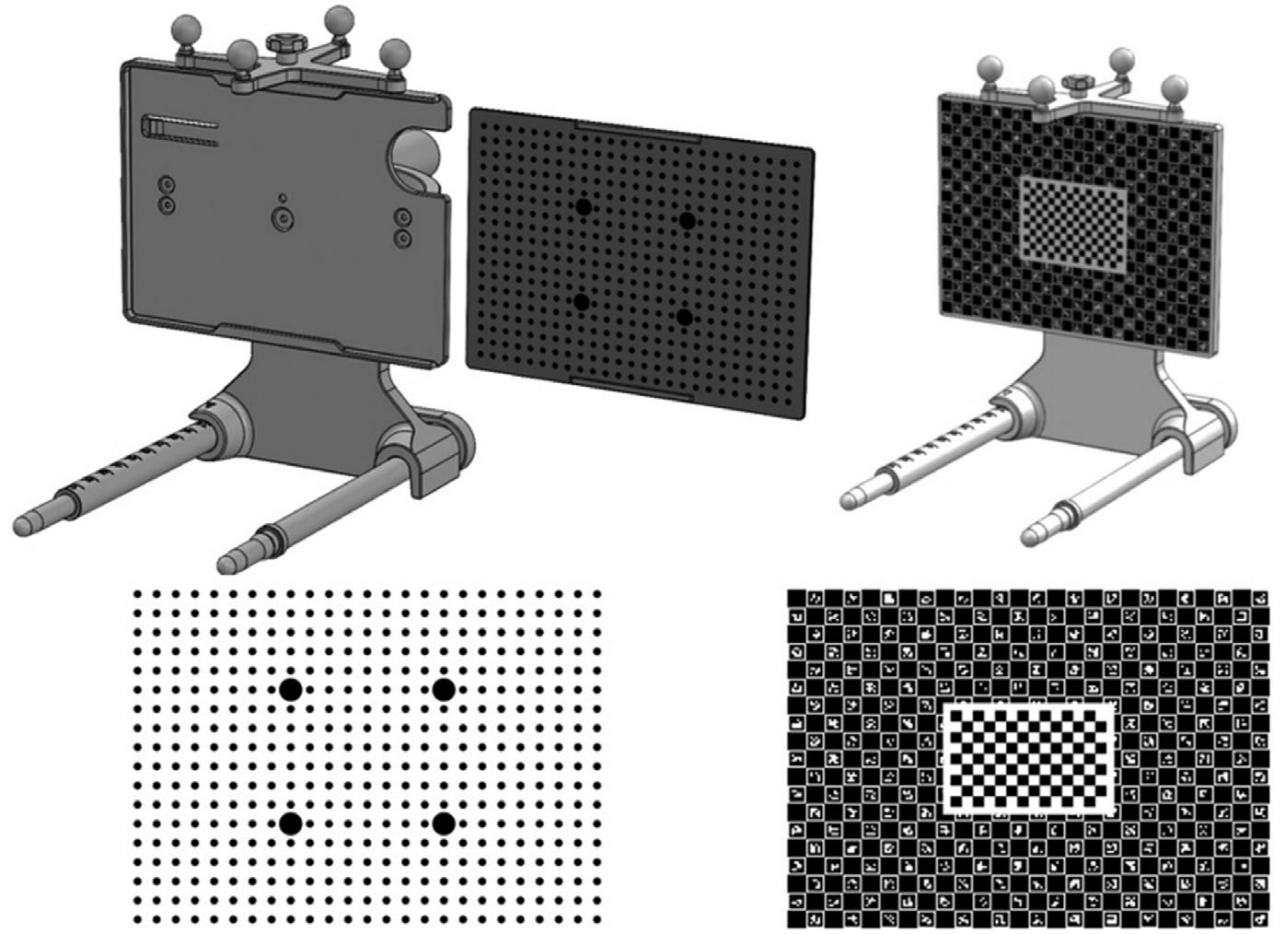
Top—laser etched removable calibration targets for dot grid and ChArUco + chessboard pattern. Bottom—frontal view of calibration patterns.

The calibration pattern itself is a 132 mm × 97 mm dot grid pattern (Figure 2), fabricated on an aluminum plate using a Violino2 Laser Cutter.^38^ This was chosen for manufacturing reasons, as the pattern can be more accurately laser etched than the alternative pattern considered, a ChArUco + Chessboard pattern, that is, a ChArUco pattern with an additional chessboard placed at its center. A standard chessboard pattern was purposefully excluded, as this requires the entire pattern to be visible for successful calibration, whereas the other patterns require only central elements to be visible, reducing potential errors and delays when operating in theater. The ChArUco pattern used in this work was laser printed and affixed to the calibration rig plate.

### 2.1 Aims

The goals of this work were to (a) compare calibration accuracy between the rig and freehand calibration; (b) assess the calibration accuracy of the manufactured dot grid pattern against ChArUco + chessboard pattern; (c) define a suitable protocol for calibration, which can be used by nonexpert staff in a clinical setting.

### 2.2 Data

A single calibration data set is defined as 10 frames of stereo data, where each frame consists of a left/right image pair, and tracking data for the laparoscope and the calibration target. Multiple sets of data were collected for each of the following scenarios:

– Freehand calibration with ChArUco + chessboard pattern (*n* = 10, 100 total frames of data)
– Freehand calibration with dot pattern (*n* = 10, 100 total frames of data)
– Rig calibration with ChArUco + chessboard pattern (*n* = 20, 200 total frames of data)
– Rig calibration with dot pattern (*n* = 20, 200 total frames of data)

**FIGURE 3:**
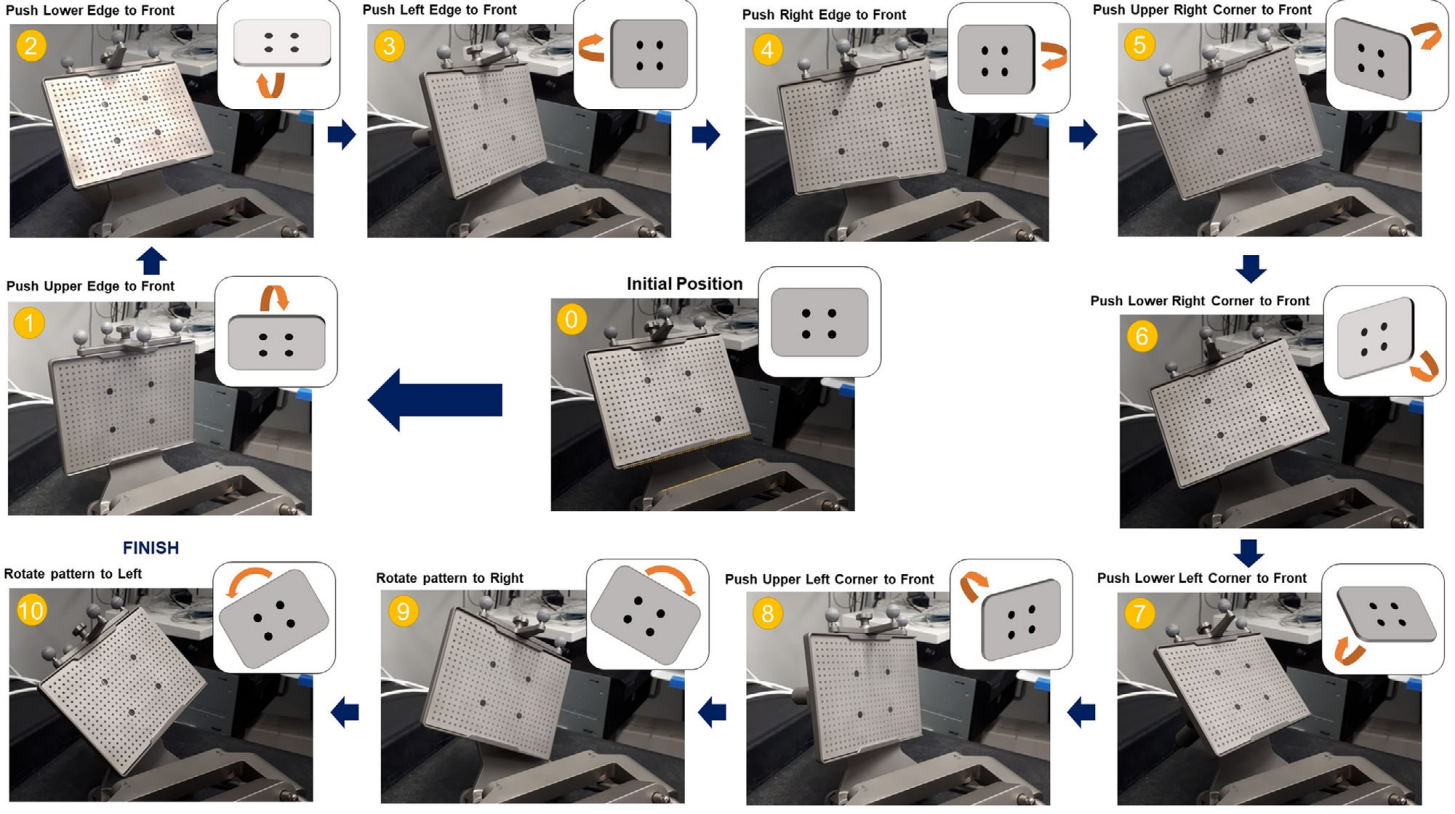
Calibration protocol used for collection of 10 calibration frames.

Data were collected using the SmartLiver^1^ software package, which is an application‐specific UI built on top of the open source scikit‐surgery libraries.^39^ A standardized 10 frame data collection protocol was established to ensure that consistent data capture can be carried out by nonexpert users (Figure 3).The choice of frames is intended to give a trade‐off between time required to collect the data, and ensuring a sufficient level of variation in the pattern position. In practice, more/fewer frames could be used for calibration if required. For freehand calibration, the user attempted to capture the same views of the calibration pattern. Data were collected in a mock operating theater, with comparable lighting to a real OR, and with the laparoscope light source activated. The laparoscope focus was not altered during data collection. An NDI Vega optical tracker was used to capture tracking data for the laparoscope and calibration target. All captured data are available online: (https://rdr.ucl.ac.uk/articles/dataset/WEISS_Laparoscope_Calibration_Study_Dataset/21930753).

### 2.3 Data processing

For comparison with typical approaches to evaluating accuracy, where all available frames of data are used for both calibration and evaluation, the following procedure, hence referred to as the “traditional” method, was carried out for each data set (10 frames):

1. Calibration pattern features were extracted from images using an appropriate point detector, implemented in the *scikit‐surgeryimage* library.
2. Left/right camera intrinsics and extrinsics were calculated individually, using OpenCV's *calibrateCamera* function, followed by stereo calibration using *stereoCalibrate*, and pose optimization using *solvePnP*.
3. Hand–eye calibration, implemented in the *scikit‐surgerycalibration* library, was carried out.
4. Stereo reprojection, stereo reconstruction, tracked stereo reprojection, and tracked stereo reconstruction error were calculated for each calibration. The mean time taken to detect calibration features in an image was also recorded, along with the time taken for the calibration algorithm to run.
5. Statistical differences between different data sets were assessed using the Welch's *t*‐test, as the sample sizes are unequal between the freehand and rig groups of data.

In order to provide a more realistic evaluation of the accuracy, involving data not used for calibration, the following method, hence referred to as the “**precalibration**” method, was used:

1. For each batch of data (Freehand ChArUco, Rig ChArUco, Rig Dots), pick one of the *n* data sets (10 frames) to act as the calibration set, solving for camera intrinsics, stereo parameters, and pose as above. If feature detection fails at any point, abort the current processing iteration and move on to the next one.
2. On each of the remaining *n*−1 data sets, perform hand–eye calibration.
3. Calculate error metrics, as above.
4. Repeat analysis *n* times, using a different data set for calibration each time, and take the mean values of error metrics. This results in (*n* * *n*−1) separate error metrics being reported.

For the purposes of this work, data processing was carried out in bulk after all data had been collected. For clinical cases, calibration can be carried out “live” in theater, using the same algorithms and software libraries, as part of the SmartLiver package. The code used for data processing is available at https://github.com/UCL/WEISS_Calibration_Study.

### 2.4 Calibration error calculation

For each calibration pattern, the 3D positions of each feature/point are known. In practice, as the calibration plates are flat, all points have a *z*‐coordinate of 0, with variation in the *x* and *y* planes only.

Stereo reprojection error is calculated by projecting the 3D model points into 2D camera space, using OpenCV's *projectPoints* function. The error is the distance, in pixels, between the reprojected points and the detected 2D points.

(1) $$[\sqrt{\frac{1}{n}\sum_{1}^{n}{\left(i_{nx}-r_{nx}\right)}^{2}+{\left(i_{ny}-r_{ny}\right)}^{2}}$$

where $i_{nx}$ and $r_{nx}$ are the *x* coordinates of the *n*th point, in the image and the reprojected data, respectively. $i_{ny}$ and $r_{ny}$ are the *y* coordinates of the *n*th point.

Stereo reconstruction error is calculated by triangulating the detected 2D image points and transforming them into the same 3D space as the model points, and measuring the distance, in millimeters, between the two sets of points.

(2) $$[\sqrt{\frac{1}{n}\sum_{1}^{n}{\left(m_{nx}-t_{nx}\right)}^{2}+{\left(m_{ny}-t_{ny}\right)}^{2}+{\left(m_{nz}-t_{nz}\right)}^{2}}$$

where $m_{nx}$ and $t_{nx}$ are the *x* coordinates in tracker space of the *n*th model points and triangulated point, respectively. $m_{ny}$ and $t_{ny}$ are the *y*‐coordinates, and $m_{nz}$ and $t_{nz}$ are the *z* coordinates.

Both of these approaches use the camera extrinsics returned by OpenCV to provide the position of the calibration target relative to the camera. For tracked reprojection/reconstrucion error, the same methods are carried out, except the pattern to camera transform is calculated using the results of the hand–eye calibration.

All code used to calculate errors can be found in the *scikit‐surgerycalibration* library, in particular the *video_calibration_metrics* module.

### 2.5 Varying number of frames used for calibration

The 10 frame protocol used in this work was selected to ensure repeatability of image capture between different calibrations, but it is not a hard requirement of the calibration system as a whole. Using fewer frames for calibration will reduce the total acquisition/processing time, at the expense of calibration accuracy. In order to investigate this trade off, calibration was repeated using fewer frames (3 and 5), where frames were randomly sampled from the 10 captured frames.

## 3 RESULTS

**FIGURE 4:**
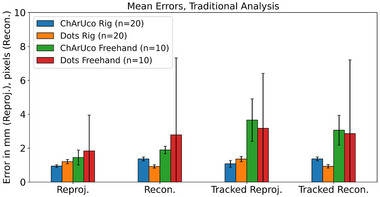
Mean errors for freehand with ChArUco pattern, rig with ChArUco pattern, and rig with dot grid pattern. Error bars indicate standard deviation. Traditional analysis method—calibration and evaluation on same data.

**FIGURE 5:**
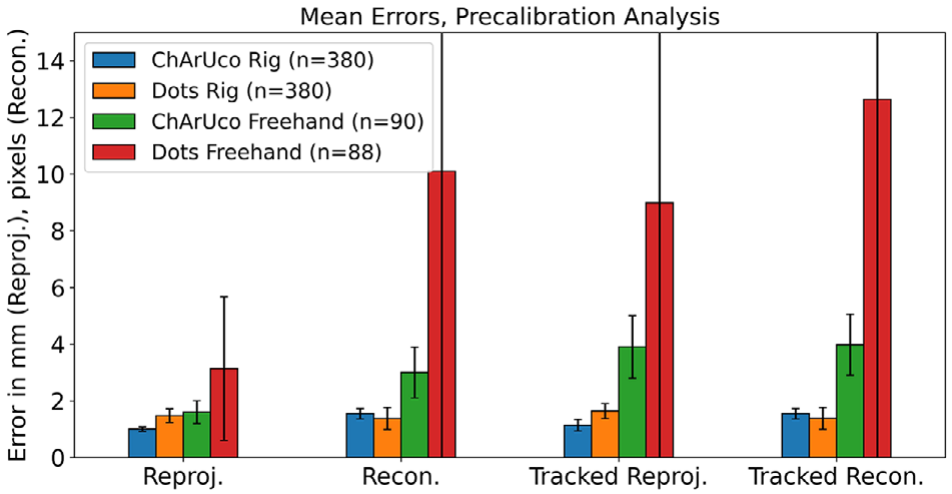
Mean errors for freehand with ChArUco pattern, rig with ChArUco pattern, and rig with dot grid pattern. Error bars indicate standard deviation. Precalibration analysis method—calibration and evaluation on different data sets. Two datasets for Dots freehand failed feature detection.

**FIGURE 6:**
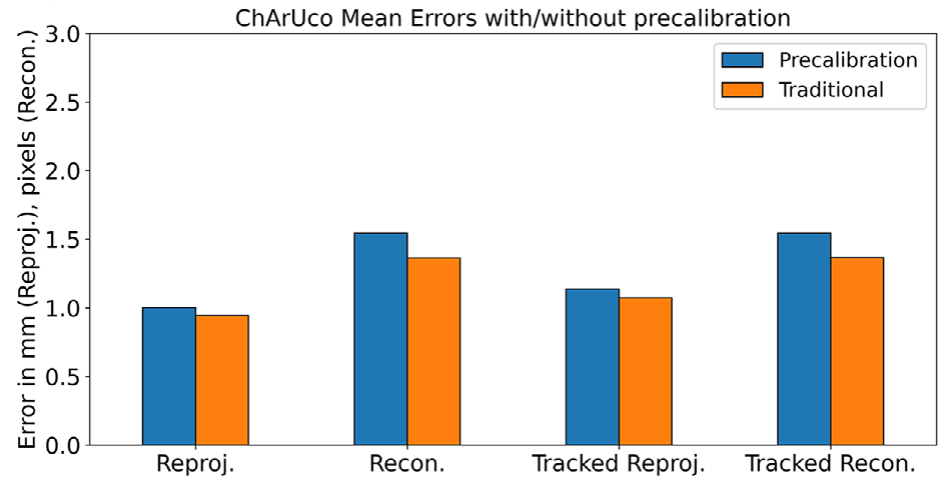
Comparison of error metrics on ChArUco pattern, for the two evaluation methods. *p*‐values: reprojection, 2e−7; reconstruction, 2e−12; tracked reprojection, 2e−2; tracked reconstruction 2e−12.

**FIGURE 7:**
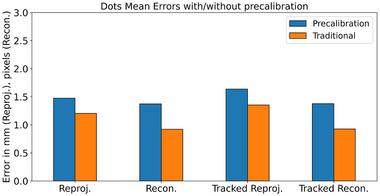
Comparision of error metrics on Dots pattern, for the two evaluation methods. *p*‐values: reprojection, 9e−15; reconstruction, 3e−26; tracked reprojection, 2e−12; tracked reconstruction 2e−25.

**FIGURE 8:**
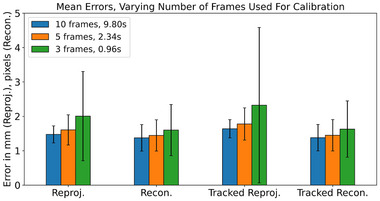
Comparison of error metrics when varying the number of frames used for calibration. All data shown from precalibraiton analysis on the dot pattern collected using calibration rig. Legend indicates the number of frames used, and the mean time taken for calibration. *p*‐values, when comparing 10 and 3 frames: reprojection, 7e−14; reconstruction, 2e−7; tracked reprojection, 1e−8; tracked reconstruction 1e−7.

Results are summarized in Figures 4, 5, 6, 7, 8 and Tables 1, 2, 3, 4. All results referred to below are for the precalibration method. As expected, this approach results in slightly higher error metrics than calibrating and evaluating on the same data in the traditional method (Figures 6 and 7).

**TABLE 1:** Mean errors, precalibration method.

Method	Reprojection	Reconstruction	Tracked reprojection	Tracked reconstruction
Rig ChArUco	1.00 (0.08)	1.54 (0.12)	1.14 (0.20)	1.54 (0.12)
Rig Dots	1.47 (0.13)	1.37 (0.10)	1.64 (0.16)	1.38 (0.10)
Freehand ChArUco	1.60 (0.43)	3.00 (0.22)	3.91 (1.25)	3.80 (0.88)
Freehand Dots	3.14 (2.11)	10.10 (4.54)	8.99 (3.24)	12.64 (4.34)

Reprojection errors in millimeters, reconstruction errors in pixels. Standard deviation is given in brackets.

**TABLE 2:** Mean errors, calibration, and evaluation on the same data (traditional method).

Method	Reprojection	Reconstruction	Tracked reprojection	Tracked reconstruction
Rig ChArUco	0.94 (0.08)	1.36 (0.12)	1.07 (0.20)	1.37 (0.12)
Rig Dots	1.20 (0.13)	0.93 (0.10)	1.35 (0.16)	0.93 (0.10)
Freehand ChArUco	1.45 (0.43)	1.89 (0.22)	3.65 (1.25)	3.06 (0.88)
Freehand Dots	1.83 (2.11)	2.78 (4.54)	3.13 (3.23)	2.85 (4.34)

Reprojection errors in millimeters, reconstruction errors in pixels. Standard deviation is given in brackets.

**TABLE 3:** Mean feature detection and mean total calibration time, in seconds, precalibration method.

Method	Feature detection time	Calibration time
Rig ChArUco	1.27 (0.06)	16.87 (6.94)
Rig Dots	0.87 (0.05)	9.17 (2.47)
Freehand ChArUco	1.22 (0.28)	27.83 (15.12)
Freehand Dots	0.97 (0.13)	25.45 (7.15)

Standard deviation is given in brackets.

**TABLE 4:** Mean feature detection and mean total calibration time, in seconds, traditional method.

Method	Feature detection time	Calibration time
Rig ChArUco	1.32 (0.06)	15.52 (6.95)
Rig Dots	0.94 (0.06)	9.80 (2.48)
Freehand ChArUco	1.32 (0.28)	30.06 (15.12)
Freehand Dots	1.03 (0.13)	30.13 (7.15)

Standard deviation is given in brackets.

### 3.1 Freehand versus rig

Use of the calibration rig and dot pattern significantly reduced mean errors for reprojection (1.47 mm [SD 0.13] vs. 3.14 mm [SD 2.11], *p*‐value 1e−8), reconstruction (1.37 px [SD 0.10] vs. 10.10 px [SD 4.54], *p*‐value 6e−7), and tracked reconstruction (1.38 mm [SD 0.10] vs. 12.64 mm [SD 4.34], *p*‐value 1e−6) when compared with freehand calibration, with no significant change in reconstruction error (Figure 5 and Table 1).

Use of the calibration rig and ChArUco pattern also produced statistically significant decreases in error. Reprojection (1.00 mm [SD 0.08] vs. 1.60 mm [SD 0.43], *p*‐value 1e−24), reconstruction (1.54 px [SD 0.12] vs. 3 px [SD 0.22], *p*‐value 6e−27), tracked reprojection (1.14 mm [SD 0.20] vs. 3.91 mm [SD 1.25], *p*‐value 1e−40), and tracked reconstruction (1.54 mm [SD 0.12] vs. 3.80 mm [SD 0.88], *p*‐value 4e−37) (Figure 5 and Table 2).

### 3.2 ChArUco versus dots

When using the rig, the ChArUco pattern produced a lower reprojection error (1.00 mm [SD 0.08] vs. 1.47 mm [SD 0.13], *p*‐value = 3e−14) and tracked reprojection error (1.14 mm [SD 0.20] vs. 1.64 mm [SD 0.16], *p*‐value = 3e−14) compared with the dot pattern, while the dot pattern produced a lower reconstruction (1.37 px [SD 0.10] vs. 1.54 px [SD 0.12], *p*‐value = 1e−14) and tracked reconstruction error (1.38 px [SD 0.10] vs. 1.54 px [SD 0.12], *p*‐value = 1e−13) (Figure 5 and Table 2).

### 3.3 Runtime

Total calibration time, measured as the time taken for the calibration algorithm to run, once all data are collected (Table 3), was higher for freehand calibration than using the rig, for the same calibration pattern. Dot calibration was also faster than ChArUco calibration (9.17 s [SD 2.42] vs. 16.87 s [SD 6.94] per calibration, *p*‐value 1.87e−69). The main contribution to the calibration time is the hand–eye optimization step, which uses a least squares optimizer to minimize the associated cost function. Total run time can be reduced if needed by relaxing the stopping criteria associated with the optimizer. The time required to collect calibration data is also reduced when using the rig, although this was not specifically measured. It is quicker to adjust the angle of the calibration target relative to the scope in the rig‐ than it is to manually move the laparoscope into the correct position relative to the target. Freehand data collection is also more likely to capture bad/blurry images, due to the difficulty of keeping the laparascope steady.

### 3.4 Varying number of frames used for calibration

As expected, reducing the number of frames used for calibration increases the overall errors across all metrics (Figure 8), while also decreasing the time required for processing. Reprojection (1.48 mm for 10 frames vs. 2.00 mm for 3 frames, *p*‐value 7e−14), reconstruction (1.37 px vs. 1.60 px, *p*‐value 2e−7), tracked reprojection (1.64 mm vs. 2.32 mm, *p*‐value 1e−8), tracked reprojection (1.38 px vs. 1.63 px, *p*‐value 1e−7), and mean processing time (9.80 s vs. 0.96 s, *p*‐value 1e−250).

## 4 DISCUSSION

The use of the calibration rig has been demonstrated to improve the accuracy of the calibration, across all error metrics used (Figures 4 and 5, and Tables 1 and 2), and to decrease the data processing time (Tables 3 and 4). The authors' attribute this to a number of factors. First, there is a much greater level of control over the rotation/angle of the calibration target relative to the laparoscope. When performing freehand calibration, the user must estimate the correct positions in which to place the laparoscope. Second, as the laparoscope and pattern are held in place, motion artefacts are eliminated, whereas manually holding the laparoscope still enough to capture data is far more challenging. Third, the rig method allows for greater reproducibility between different calibrations; even a highly experienced user will find it difficult to maneuvre the laparoscope to a fixed distance/rotation relative to the pattern on every occasion. This means that a “bad” calibration, where there is an insufficient variety of positions captured, is more likely to occur.

Aside from quantitative improvements, there are also qualitative improvements for the user(s). Maneuvring the laparoscope when performing freehand calibration can be challenging physically, requiring the user to change positions, manage trailing cables, engage/disengage a clamp to hold the laparoscope still, while also monitoring the laparascope feed to ensure that the desired position has been achieved.

While the use of the calibration rig does introduce some additional equipment into the clinical workflow, this is a one‐off step that can be performed prior to surgery beginning, by a member of the nursing/support staff, and would not directly interfere with the surgeon's workflow.

### 4.1 Future work

While the data for this work were collected by a member of the engineering team, in the longer term, it will be necessary to have a member of the clinical team carry out the calibration. While work has previously been carried out in which clinical staff were asked to assemble and use the calibration rig, as part of a wider study on Human Computer Interaction,^40^ and also work comparing the overall usability and performance of the SmartLiver system for registration, as of yet there has been no formal study directly comparing the calibration results from novice users, with experienced users of the rig. While the authors estimate anecdotally that a user can become competent in following the protocol after 5–10 attempts, a future study where the learning curve of novice users, both engineers and surgeons, is formally assessed will provide further guidance to inform clinical practice.

## 5 CONCLUSION

The following conclusions can be made:

(a) The use of a calibration rig results in a statistically significant decrease in the calibration error metrics, when compared with freehand calibration (Figures 4 and 5).
(b) Based on error metrics, there is no clear reason to favor either the dot or the ChArUco + chessboard pattern. In terms of processing time required, the dot pattern is quicker (Table 3). As such, the choice to manufacture the dot pattern is validated.

In terms of identifying the preferred approach for calibrations within the operating theater, the calibration rig should be used, alongside the dot pattern, due to ease of manufacture, with 10 frames of data providing sufficient variation to minimize errors.

## CONFLICT OF INTEREST STATEMENT

The authors declare no conflicts of interest.
